# Dose response effects of a caffeine-containing energy drink on muscle performance: a repeated measures design

**DOI:** 10.1186/1550-2783-9-21

**Published:** 2012-05-08

**Authors:** Juan Del Coso, Juan José Salinero, Cristina González-Millán, Javier Abián-Vicén, Benito Pérez-González

**Affiliations:** 1 Exercise Physiology Laboratory, Camilo José Cela University, C/ Castillo de Alarcon, 49, Villafranca del Castillo 28692, Spain

**Keywords:** Caffeine, Sports nutrition, Force production, Exercise, Energy expenditure

## Abstract

**Background:**

Energy drinks have become the most used caffeine-containing beverages in the sport setting. The aim of this study was to determine the effects of two doses of a caffeine-containing energy drink on muscle performance during upper- and lower-body power-load tests.

**Methods:**

In a randomized order, twelve active participants ingested 1 and 3 mg of caffeine per kg of body weight using a commercially available energy drink (Fure®, ProEnergetics) or the same drink without caffeine (placebo; 0 mg/kg). After sixty minutes, resting metabolic rate, heart rate and blood pressure were determined. Then, half-squat and bench-press power production with loads from 10 to 100% of 1 repetition maximum was determined using a rotator encoder.

**Results:**

In comparison to the placebo, the ingestion of the caffeinated drink increased mean arterial pressure (82 ± 7 < 88 ± 8 ≈ 90 ± 6 mmHg for 0 mg/kg, 1 mg/kg, 3 mg/kg of caffeine, respectively; *P* < 0.05) and heart rate (57 ± 7 *<* 59 ± 8 *<* 62 ± 8 beats/min, respectively; *P* < 0.05) at rest in a dose response manner, though it did not affect resting metabolic rate. While the ingestion of 1 mg/kg of caffeine did not affect maximal power during the power-load tests with respect to the placebo, 3 mg/kg increased maximal power in the half-squat (2554 ± 167 ≈ 2549 ± 161 < 2726 ± 167 W, respectively; *P* < 0.05) and bench-press actions (349 ± 34 ≈ 358 ± 35 < 375 ± 33 W, respectively; *P* < 0.05).

**Conclusions:**

A caffeine dose of at least 3 mg/kg in the form of an energy drink is necessary to significantly improve half-squat and bench-press maximal muscle power.

## Introduction

Caffeine (1,3,7- trimethylxanthine) is a natural alkaloid present in the leaves, fruits and seeds of various plants (coffee, kola, tea, mate, etc); yet it can also be artificially synthesized in the laboratory. This dual origin of caffeine has turned this substance into the most frequently ingested drug in the world [1] since it is present in foods and drinks (chocolate, coffee, and soft drinks), dietary supplements, and over-the-counter medications. In the sports setting, caffeine is consumed prior to competing by 74% of elite national and international athletes, based on the caffeine concentration found in the urine samples obtained for doping analysis [2]. The current popularity of caffeine in sports is associated with the physical benefits derived from its ingestion in a wide variety of sports activities [3] and the removal of caffeine from the list of prohibited substances published by the World Anti-doping Agency in 2004 [4].

The ingestion of pure anhydrous caffeine in capsules or powder has been the most typical experimental setting to investigate the effects of caffeine on sports performance [5]. The ingestion of 3 to 9 mg of caffeine per kg of body mass has been repeatedly shown as ergogenic in several exercise activities [6-12]. Doses of caffeine as high as 13 mg/kg [13] or as low as 2 mg/kg [14] have been reported to have an ergogenic effect of a similar magnitude to the one observed with the typical 3-to-9 mg/kg doses. However, the ingestion of 1 mg/kg of caffeine has failed to improve endurance performance [14].

As opposed to caffeine capsules, the newly created caffeine-containing energy drinks have become the most used means for caffeine intake in the sports population [15-17]. These energy drinks typically contain moderate amounts of caffeine (32 mg per 100 mL of product) in addition to carbohydrates, taurine, glucoronolactone and B-group vitamins [18]. The effects of these energy drinks on physical performance are diverse and the scientific literature scarce. The intake of one serving of an energy drink (250 mL, equivalent to ~1 mg of caffeine per body weight) did not enhance maximal oxygen uptake during a maximal effort test [19], peak power during three repetitions of the Wingate test [20,21] or running velocity during 24 “all-out” sprints [22]. However, one serving of an energy drink improved reaction time, alertness and aerobic and anaerobic performance tests [23]. The ingestion of two servings of a caffeine-containing energy drink (equivalent to ~2 mg of caffeine per kg) reduced the time necessary to complete a cycling time trial [24] but did not increase the time-to-exhaustion during a running test [25]. Finally, the ingestion of 3 mg/kg of caffeine in the form of an energy drink increased jump height, sprint velocity and running distance covered during a simulated game [26]. Thus, more investigations are necessary to reveal whether the effects of caffeine-containing energy drinks on sports performance are dose-dependent.

The ergogenic properties of caffeine on muscle power-strength performance have been less well studied [12] while the outcomes are confusing since the investigations have used different performance protocols, caffeine dosing and participants’ training status [27]. In a recent meta-analysis, Warren et al. [28] reported strong evidences regarding the ergogenic effects of caffeine on leg muscle strength, though unlike effects found in other muscle groups. Nevertheless, these physical benefits were present when ingesting ~ 6 mg/kg of anhydrous caffeine. Still, to our knowledge, no investigation has focused on the dose–response effects of caffeine-containing energy drinks on muscle strength and power. The aim of this study was to investigate the effects of 1 and 3 mg of caffeine per kg of body weight via an energy drink on muscle performance during upper and lower body power-load tests.

## Methods

### Subjects

Twelve healthy and active participants volunteered to participate in this study. The study included three women who were always tested in the luteal phase. Subjects had a mean ± SD age of 30 ± 7 yrs, body mass of 69 ± 10 kg, height of 173 ± 8 cm and body fat percentage of 18 ± 8%. Their one-repetition maximum (1 RM) in concentric actions was on average 94.3 ± 16.5 kg for the half-squat and 46.3 ± 13.9 kg for the bench-press. The participants had not been involved in body resistance-training programs 3 months prior to the study and they had no physical limitations or musculoskeletal injuries that could affect the results of the study. In addition, participants were non-smokers whilst they were light caffeine consumers (< 60 mg per day, ~ 1 cup of coffee).

### Ethics statement

Participants were fully informed of any risks and discomforts associated with the experiments before giving their informed written consent to participate. The study was approved by the Camilo José Cela University Review Board in accordance with the latest version of the Declaration of Helsinki.

### Pre-experimental procedures

One week before the experimental trials, participants underwent a physical examination to ensure that they were in good health. After that, participants were nude weighed (± 50 g, Radwag, Poland) to individualize caffeine doses, and their body fat composition was calculated using bioimpedance (BC-418, Tanita, Japan). After a standardized warm-up, all subjects performed a maximal strength test with increasing loads to determine their 1 RM in the concentric phases of half-squat and bench-press actions. For these tests, participants started from knees (half-squat) and arms (bench-press) flexed at 90° to reach full limb extension against the load which consisted of a bar and weight plates added to the bar. Four to five attempts with progressive loads were performed for each action until the subjects were unable to attain 180° limb extensions. The last acceptable attempt with the highest possible load was determined as 1 RM, expressed in kg. The day before these tests and from this pre-experimental session to the beginning of experimental trials, participants were instructed to avoid strength training or strenuous exercise.

### Experimental design

A double-blind, placebo controlled and randomized experimental design was used in this study. Each participant performed 3 experimental trials at the same time of day and under laboratory controlled conditions (21°C dry temperature; 30% relative humidity). On one occasion, participants ingested 3 mg of caffeine per kg of body mass (3 mg/kg; 207 ± 30 mg) by means of 250 mL of a commercially available caffeine-containing energy drink (Fure®, Proenergetics®, Spain). On another occasion, participants ingested the same amount of energy drink but with a lower caffeine concentration to provide 1 mg/kg of caffeine to each participant (1 mg/kg; 69 ± 10 mg). On the third occasion, participants ingested the same amount of energy drink but with no caffeine content (placebo; 0 mg/kg). At the request of the experimenters, the manufacturer provided the same energy drink with different amounts of caffeine to achieve a similar taste and appearance. The energy drinks also contained taurine (2000 mg) sodium bicarbonate (500 mg), L-carnitine (200 mg) and maltodextrin (705 mg). However, the trials differed only in the amount of caffeine administrated. The beverages were ingested 60-min before the onset of the experimental trials to allow complete caffeine absorption [29] and they were provided in opaque plastic bottles to avoid identification. The order of the experimental trials was randomized and counterbalanced. An alphanumeric code was assigned to each trial to blind participants and investigators to the drink tested. This code was unveiled after the analysis of the variables. The experimental trials were separated by at least 48-h to allow complete caffeine washout.

### Resting measurements

The day before each experimental trial, participants refrained from strenuous exercise and adopted a similar diet and fluid intake regimen. Participants were encouraged to withdraw from all dietary sources of caffeine (coffee, cola drinks, chocolate, etc) and alcohol for 48 hours before testing. In addition, participants were instructed to have a light meal at least two hours before the onset of the experimental trials. Participants arrived at the laboratory and drank the beverage assigned for the trial. They then dressed in a T-shirt, and shorts and a heart rate belt (Polar®, Finland) was attached to their chest. After that, they rested supine for 60 minutes to allow caffeine absorption. Later, gas exchange data were collected continuously for 15 min using an automated breath by breath system (Metalyzer, Cortex, Germany) to calculate resting oxygen uptake (VO_2_) and carbon dioxide production (VCO_2_). During the last 3-min of each collection period, the gas exchange data were averaged to achieve a representative value for the energy drink. Resting energy expenditure (cal/min) was calculated as (3.869 × VO_2_) + (1.195 × VCO_2_), where VO_2_ and VCO_2_ are in L/min [30]. Certified calibration gases (16.0% O_2_; 5.0% CO_2_, Cortex, Germany) and a 3-L syringe were used to calibrate the gas analyzer and the flowmeter before each trial. During this period, resting heart rate was also recorded. Next, systolic blood pressure and fourth phase diastolic blood pressure (DBP) were measured on the left arm using a manual sphingomanometer (Riester, Germany) while the participant lay supine. Mean arterial pressure (MAP) was calculated as MAP = diastolic blood pressure + 0.33 (systolic blood pressure – diastolic blood pressure). This measurement was always obtained by the same practiced experimenter who was also unaware of the drink being tested.

### Power-load tests

After the resting measurements, participants performed a standardized warm-up that included 10-min running and leg and arm extensions with submaximal loads. Next, the power-load relationship in the half-squat and bench-press actions was tested concentrically by using relative loads from 10 to 100% of 1 RM (10% increments). In the half-squat test, the shoulders were in contact with the bar, the starting knee angle was 90° and security belts were used by all the participants to keep the trunk straight. The resistance was supported by the bottom stops of the measurement device to isolate concentric muscle actions. On command, the participant performed a concentric leg extension as fast as possible against the resistance provided by the bar and the weight plates added to the bar. We set a 2-min resting period between repetitions. In the bench-press test, the bar was positioned above the participant’s chest to maintain the arms flexed at 90°. On a verbal command, participants performed a concentric arm extension as fast as possible, while no bouncing or arching of the back was permitted.

These power-load tests were always performed in machinery in which the resistance bar was attached at both ends with linear bearings on two vertical bars, thus allowing only vertical movements of the bar (Multipower, Technogym, Spain). This machinery allowed participants to jump (in half-squat) or release the bar (in bench press) when feasible, in order to avoid deceleration during the concentric movements. During each repetition, velocity (in m/s), acceleration (in m/s^2^) and power (in W) were recorded at 1000 Hz by linking a rotator encoder (Isocontrol, Spain) to the end of the bar. Customized software (JLML, Spain) was used to calculate maximal force production (resistance × acceleration) and maximal power output (force × velocity) for each repetition. Standardized encouragement and feedback were given to the participants by the same experimenter, who was blind to the treatments, to produce maximal effort in each repetition.

### Side-effects evaluation

The following morning of each experimental trial, participants responded to a telephone survey about sleep quality, nervousness, gastrointestinal problems and other discomforts associated with the energy drinks ingestion. This survey included 8 items on a yes/no scale. This questionnaire was based on previous publications about side effects derived from the ingestion of caffeine [31,32].

### Statistical analysis

Resting metabolic rate, heart rate and blood arterial pressures were analyzed by using one-way analyses of variance (ANOVA) with repeated measures (caffeine dose). The power-load and force-velocity relationships were compared using two-way ANOVA with repeated measures (caffeine dose × load) to determine differences within caffeine content of the drinks. After a significant *F* test, differences among means were identified using the Bonferroni *post hoc* procedure. To analyze the effects of the energy drinks on side-effects we used a non-parametric test for dichotomic variables and related samples (Cochran test). We used the coefficient of determination (R^2^) to assess the association between force and velocity. The significance level was set at *P* < 0.05. The results are presented as means ± SD.

## Results

### Resting measurements

In comparison to the placebo, the ingestion of 1 mg/kg and 3 mg/kg of caffeine using an energy drink increased resting systolic blood pressure, diastolic blood pressure, mean arterial pressure and heart rate in a dose–response manner (Table 1; *P* < 0.05). On the other hand, these caffeine doses did not affect resting energy expenditure, mechanical ventilation or respiratory exchange ratio (Table 1).

**Table 1:** Resting values for metabolic and cardiovascular variables one hour after the ingestion of 1 and 3 mg/kg of caffeine using a caffeinated energy drink or the same drink without caffeine (0 mg/kg). Data are mean ± SD for 12 participants

**Resting values**	**0 mg/kg**	**1 mg/kg**	**3 mg/kg**
Energy expenditure (cal/min)	1.4 ± 0.2	1.4 ± 0.3	1.4 ± 0.3
Mechanical ventilation (L/min)	7.7 ± 1.5	8.2 ± 1.5	8.2 ± 1.5
Respiratory Exchange Ratio	0.84 ± 0.03	0.87 ± 0.03	0.85 ± 0.04
Systolic blood pressure (mmHg)	112 ± 12	119 ± 10*	118 ± 19*
Diastolic blood pressure (mmHg)	68 ± 5	73 ± 8*	76 ± 5*†
Mean arterial pressure (mmHg)	82 ± 7	88 ± 8*	90 ± 6*
Heart rate (beats/min)	57 ± 7	59 ± 8*	61 ± 8*†

* Different from 0 mg/kg (*P* < 0.05). † Different from 1 mg/kg (*P* < 0.05).

### Power-load test

**Figure 1:**
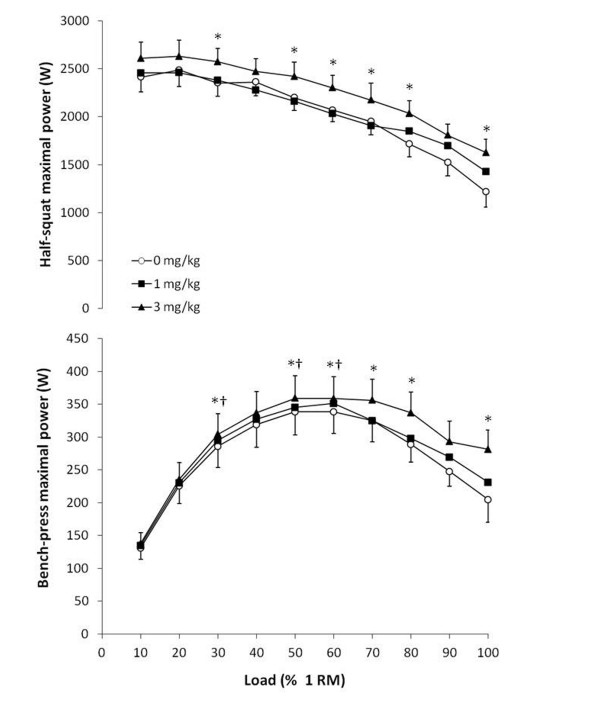
**Power-load curves for half-squat and bench-press concentric actions one hour after the ingestion of 1 and 3 mg/kg of caffeine using a caffeinated energy drink or the same drink without caffeine (0 mg/kg).** Data are mean ± SD for 12 participants. * 3 mg/kg different from 0 mg/kg (*P* < 0.05). † 3 mg/kg different from 1 mg/kg (*P* < 0.05).

Maximal power output in the half-squat power-load test was 2554 ± 167 W after 0 mg/kg, similar to 2549 ± 161 W after 1 mg/kg and both less than after 3 mg/kg (2726 ± 166 W; *P* < 0.05). The same differences were found in the bench-press power load-test (349 ± 34 ≈ 359 ± 35 < 375 ± 33 W, respectively; *P* < 0.05). Figure 1 depicts the power-load curves for the half-squat and bench-press concentric actions after the ingestion of 0 mg/kg, 1 mg/kg and 3 mg/kg of caffeine in the form of energy drinks. In the half-squat, the ingestion of 3 mg/kg of caffeine moved the curve upwards in comparison to 0 mg/kg, and it significantly increased muscle power output at 30, 50, 60, 70, 80 and 100% 1RM (*P* < 0.05). In the bench-press action, 3 mg/kg of caffeine also moved the curve upwards and it significantly increased power output at 30, 50, 60, 70, 80 and 100% 1RM (*P* < 0.05). Although the ingestion of 1 mg/kg tended to increase power at high loads (Figure 1), it did not reach statistical significance in the half-squat or bench press at any load.

### Force-velocity relationship

**Figure 2:**
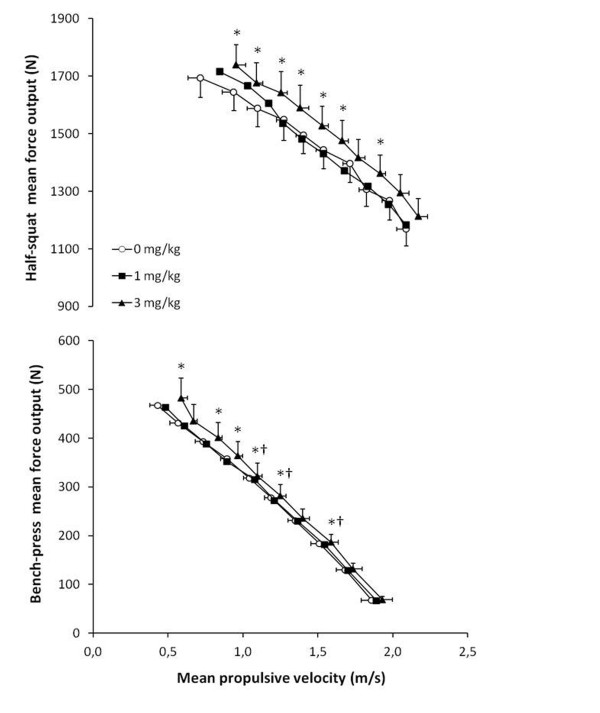
**The force-velocity relationship for half-squat and bench-press concentric actions one hour after the ingestion of 1 and 3 mg/kg of caffeine using a caffeinated energy drink or the same drink without caffeine (0 mg/kg).** Data are mean ± SD for 12 participants. * 3 mg/kg different from 0 mg/kg (*P* < 0.05). † 3 mg/kg different from 1 mg/kg (*P* < 0.05).

Figure 2 illustrates the relationship between force production and mean propulsive velocity attained at each repetition of the power-load tests. In comparison to 0 mg/kg, the ingestion of 3 mg/kg of caffeine moved the force-velocity curve upwards and rightwards in both the half-squat and bench press (*P* < 0.05). The equations of the best fit line generated with these data and the coefficient of determination R^2^ are presented in Table 2. All the R^2^ values for the best fit lines were higher than 0.98, which means a high correlation between the outcomes and their predicted values. Although the slopes of each best fit line were similar, the Y-axis intercept with the ingestion of 3 mg/kg of caffeine was considerably increased in comparison to 0 mg/kg, since it was 2157 *vs* 1966 N in the half-squat and 649 *vs* 596 N in the bench-press for 3 mg/kg and 0 mg/kg, respectively. Since the Y-axis intercept is attained with a velocity equal to 0 m/s, these data indicate that 3 mg/kg of caffeine would also enhance isometric force production.

**Table 2:** **Best fit line equations and coefficients of determination (R**^**2**^**) for the force-velocity relationships in half-squat and bench press concentric actions one hour after the ingestion of 1 and 3 mg/kg of caffeine using a caffeinated energy drink or the same drink without caffeine (0 mg/kg). Data are mean ± SD for 12 participants**

	**0 mg/kg**	**1 mg/kg**	**3 mg/kg**
Half-squat	−380x + 1966	−439x + 2093	−430x + 2157
R^2^	0.98	0.99	0.99
Bench press	−278x + 596	−275x + 600	−297x + 649
R^2^	0.99	0.99	0.99

### Frequency of the side-effects derived from energy drink consumption

As shown in Table 3, the experimental protocol with the ingestion of the placebo energy drink (0 mg/kg) produced residual side-effects such as headache, muscle soreness, increased vigor/activeness, insomnia, and increased urine production, all of them with a frequency lower than 27%. The ingestion of an energy drink with 1 mg/kg of caffeine produced similar frequencies of side effects to the ingestion of the placebo drink. The ingestion of 3 mg/kg of caffeine in the form of an energy drink tended to increase the frequency of abdominal/gut discomfort, the incidence of tachycardia and heart palpitations and perceived anxiety, in comparison to the placebo. In addition, 3 mg/kg of caffeine tended to increase the feeling of vigor and activeness in comparison to the placebo drink in the following hours after the ingestion of the drink.

**Table 3:** Side-effects resulting from the ingestion of 1 and 3 mg/kg of caffeine using a caffeinated energy drink or the same drink without caffeine (0 mg/kg)

**Item**	**0 mg/kg**	**1 mg/kg**	**3 mg/kg**
Headache	8%	17%	8%
Abdominal/gut discomfort	0%	0%	17%
Muscle soreness	17%	17%	17%
Increased vigor/activeness	17%	8%	58%*
Tachycardia and heart palpitations	0%	0%	17%
Insomnia	17%	8%	25%
Increased urine production	8%	8%	25%
Increased anxiety	0%	8%	8%

* Different from 0 mg/kg (*P* < 0.05).

## Discussion

The purpose of this study was to examine the effects of a caffeine-containing energy drink with a dose of 1 or 3 mg/kg of caffeine on muscle performance during half-squat and bench-press exercises. Findings indicate that the ingestion of the energy drink with 1 mg/kg of caffeine was not enough to raise the power output or to modify the force-velocity association during 10-to-100% 1RM power-load tests. However, the ingestion of an energy drink with 3 mg/kg of caffeine increased maximal power output by 7 ± 4% in the half-squat and by 7 ± 2% in the bench-press, in comparison to the ingestion of a placebo energy drink (*P* < 0.05). In addition, 3 mg/kg of caffeine moved the relationship found between the force production and velocity upwards in both the half-squat and the bench press. Thus, an energy drink with at least 3 mg/kg of caffeine is necessary to significantly enhance muscle performance.

Apart from Seidl et al. [33] who investigated the effects of an energy drink on cognitive performance, the first authors to investigate the outcomes of caffeine-containing energy drinks on physical performance were Alford and co-workers [23]. Since then, a small number of studies have been geared to examining the effects of caffeine-containing-energy drinks on physical performance or sports tasks, mainly because of the relative novelty of these beverages [19-25,34]. Most of them have used the most popular energy drink, Red Bull®, which contains 80 mg of caffeine per 250 mL of product (one serving). When the investigations included the comparison between a placebo beverage and one serving of the energy drink, equivalent to ~1 mg of caffeine per kg of body weight, most [19-22], but not all studies [23], failed to find a better physical performance derived from the energy drink ingestion. On the other hand, the ingestion of two or three servings of energy drink (equivalent to ~2-3 mg of caffeine per kg) improved [24,34] or tended to improve [25] physical performance. These outcomes combined with the results of the present investigation suggest that the physical benefits attributed to caffeine-containing energy drinks are present with at least 3 servings, equivalent to ~3 mg/kg of caffeine.

The effects of caffeine ingestion on muscle strength have been previously investigated during the realization of either isometric maximal voluntary contractions (MVC) or isotonic 1 RM tests [12]. Overall, the ingestion of ~6 mg/kg of caffeine raised maximal force production during both assessments, while lower caffeine doses have not been extensively studied (see review [28]). Regarding muscle power production and caffeine ingestion, most studies have used a 4–30 s maximal cycling test. In these studies, the results are confusing since ~6 mg/kg of caffeine increased [6,35-37] or did not changed [38-43] maximal cycling power with similar 3-to-7 mg/kg caffeine doses. The experimental design used for the present investigation contains some novelties in comparison to previous studies about caffeine and muscle performance.

First, we have selected a power-load test to assess muscle performance after caffeine ingestion instead of single-resistance trials (i.e., MVC, 1RM, Wingate test, etc). This test includes maximal concentric contractions over a wide range of resistances and thus, it allows a better identification of maximal power and strength production. Similar power-load tests have been successfully used to assess the effect of training [44] and age [45] on muscle performance. Second, we have used two doses of caffeine to assess the dose–response benefits of this substance on muscle performance. These doses (1 and 3 mg/kg) were chosen based on previous publications on endurance performance tests in which the ingestion of 3 to 9 mg/kg of caffeine produced comparable benefits, while 1 mg/kg was found to be non ergogenic [7,14]. Third, we have measured the effects of caffeine ingestion on upper-body and lower-body exercises. It has been suggested that lower-body muscles are more sensitive to caffeine ingestion due to their lower activation level [28]. With this experimental design, we can conclude that caffeine increases both maximal muscle strength and muscle power even with a dose of 3 mg/kg. In addition, the effects of caffeine on lower-body and upper-body muscles were alike.

**Figure 3:**
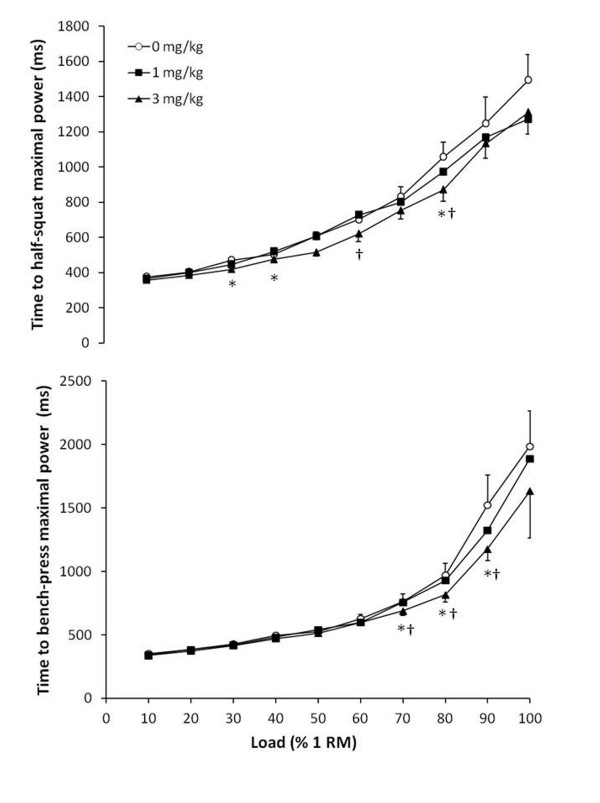
**Time to maximal power output during half-squat and bench-press concentric actions one hour after the ingestion of 1 and 3 mg/kg of caffeine using a caffeinated energy drink or the same drink without caffeine (0 mg/kg).** Data are mean ± SD for 12 participants. * 3 mg/kg different from 0 mg/kg (*P* < 0.05). † 3 mg/kg different from 1 mg/kg (*P* < 0.05).

Originally, the ergogenic effects of caffeine on physical performance were attributed to an enhancement of muscle fat oxidation and thus to a better glycogen sparing capacity derived from the intake of this substance [46]. However, caffeine has been found to be ergogenic in short-term activities where increased fat oxidation and/or carbohydrate sparing capacities are not the limiting factor for performance [27]. More recently, it has been found in animal models that caffeine may directly affect the muscle via enhanced Ca^++^ release from the sarcoplasmic reticulum [47] or via enhanced motor unit recruitment by inhibiting adenosine actions on the central nervous system [48]. In a previous study with humans, we found that 6 mg/kg of caffeine improved knee extensor muscle strength and cycling power production due to a higher voluntary contraction (central effects) with no effects on electrically evoked contractions (no effects on muscle contractile properties). Although we did not assess the source of the benefits found with caffeine-containing energy drinks in the present investigation, we did find the tendency for a lower time to maximal power output (Figure 3). A lower time to maximal power suggests a better intra- and inter-muscular coordination during the muscle contraction, likely mediated by improved motor unit recruitment [49].

In a recent study with 176 participants, Badillo and Medina [50] found a very good association (R^2^ = 0.98) between load and propulsive velocity during the concentric phase of the bench press exercise. The mean velocity attained with 100% 1RM was 0.2 m/s and it increased progressively to 1.4 m/s when the load was reduced to 30% 1RM. According to these data, the authors conclude that measurement of propulsive velocity can be used for training or testing as a good predictor of the relative load (% 1RM) using a regression equation [50]. In the present study, we found a similar correlation between load and propulsive velocity in both half-squat and bench-press exercises (Table 2). In addition, with the ingestion of the placebo drink, the velocities attained during the propulsive phase of the bench press at 100% and 30% 1RM were similar to the ones found by Badillo and Medina (0.4 ± 0.1 and 1.5 ± 0.1 m/s, respectively). On the other hand, the ingestion of the energy drink with 3 mg/kg of caffeine raised bench press velocity to 0.6 ± 0.1 m/s at 100% 1RM and to 1.6 ± 0.1 m/s at 30% 1RM (Figure 2), moving the association between load and velocity upwards. Thus, when using the propulsive velocity to predict the relative load that represents a given resistance, the ingestion of caffeine or caffeine-containing energy drinks might represent a source of error.

Previous studies have found that caffeine or coffee ingestion may increase resting energy expenditure by 3-7% [51,52]. However, in the present investigation with energy drinks, we did not find a thermogenic effect after the ingestion of 1 or 3 mg/kg of caffeine (Table 1). While we measured energy expenditure for 15 minutes, previous investigations have used more prolonged measurement periods (from 150 min to 24 h) to determine the thermogenic effect of caffeine. Thus, it could be necessary to enlarge the measurement period for the determination of resting energy expenditure to clarify if caffeine-containing energy drinks also raise energy expenditure. The acute ingestion of caffeine produces mild psychostimulant effects, which are thought to be the reason for its extensive use in the general population [31]. However, the ingestion of moderate-to-high amounts of this substance could also produce negative effects such as anxiety, headaches, elevated heart rate and blood pressure, increased sweating and urine production or insomnia [32]. The ingestion of an energy drink with 1 mg/kg of caffeine increased mean blood pressure by 5 ± 3 mmHg and heart rate 2 ± 3 beats per minute. However, this caffeine dose did not raise the prevalence of typical side effects in comparison to the placebo energy drink (see Table 3). The ingestion of an energy drink with 3 mg/kg of caffeine increased mean blood pressure by 8 ± 2 mmHg and heart rate by 4 ± 3 beats per minute in addition to a tendency for a higher frequency of abdominal/gut discomfort, incidence of tachycardia and heart palpitations and perceived anxiety (non significant). Therefore, it seems that caffeine-containing energy drinks, like pure caffeine ingestion, produce some minor side-effects in the subsequent hours to the ingestion. However, these side-effects would be only present with a caffeine dose of 3 mg/kg.

## Conclusions

The ingestion of a caffeine-containing energy drink equivalent to 1 mg/kg of caffeine does not produce significant ergogenic effects on muscle performance. According to our findings, a dose of energy drink at least equivalent to 3 mg/kg of caffeine is necessary to significantly improve lower-body and upper-body muscle power and strength. The ingestion of this second energy drink dose also increases heart rate, blood pressure, and tended to increase the frequency of some minor side-effects in the subsequent hours to the ingestion.

## Competing interests

The author(s) declare that they have no competing interests’.

## Author's contributions

JDC participated in the concept and design, carried out the data acquisition and was the main writer of the manuscript. JJS participated in the concept and design, carried out the data analysis and was a reviewer of the manuscript. CGM participated in the concept and design, carried out the data acquisition and was a reviewer of the manuscript. JAV participated in the data acquisition and analysis and was a reviewer of the manuscript. BPG participated in the data acquisition and analysis and was a reviewer of the manuscript. All authors read and approved the final manuscript.
